# An Evolutionary Algorithm to Personalize Stool-Based Colorectal Cancer Screening

**DOI:** 10.3389/fphys.2021.718276

**Published:** 2022-01-26

**Authors:** Luuk A. van Duuren, Jonathan Ozik, Remy Spliet, Nicholson T. Collier, Iris Lansdorp-Vogelaar, Reinier G. S. Meester

**Affiliations:** ^1^Department of Public Health, Erasmus University Medical Center, Rotterdam, Netherlands; ^2^Decision and Infrastructure Sciences, Argonne National Laboratory, Lemont, IL, United States; ^3^Econometric Institute, Erasmus University Rotterdam, Rotterdam, Netherlands

**Keywords:** colorectal cancer, personalized screening, fecal immunochemical test, screening interval, cutoff, microsimulation models, evolutionary algorithm, FIT-history

## Abstract

**Background:**

Fecal immunochemical testing (FIT) is an established method for colorectal cancer (CRC) screening. Measured FIT-concentrations are associated with both present and future risk of CRC, and may be used for personalized screening. However, evaluation of personalized screening is computationally challenging. In this study, a broadly applicable algorithm is presented to efficiently optimize personalized screening policies that prescribe screening intervals and FIT-cutoffs, based on age and FIT-history.

**Methods:**

We present a mathematical framework for personalized screening policies and a bi-objective evolutionary algorithm that identifies policies with minimal costs and maximal health benefits. The algorithm is combined with an established microsimulation model (MISCAN-Colon), to accurately estimate the costs and benefits of generated policies, without restrictive Markov assumptions. The performance of the algorithm is demonstrated in three experiments.

**Results:**

In Experiment 1, a relatively small benchmark problem, the optimal policies were known. The algorithm approached the maximum feasible benefits with a relative difference of 0.007%. Experiment 2 optimized both intervals and cutoffs, Experiment 3 optimized cutoffs only. Optimal policies in both experiments are unknown. Compared to policies recently evaluated for the USPSTF, personalized screening increased health benefits up to 14 and 4.3%, for Experiments 2 and 3, respectively, without adding costs. Generated policies have several features concordant with current screening recommendations.

**Discussion:**

The method presented in this paper is flexible and capable of optimizing personalized screening policies evaluated with computationally-intensive but established simulation models. It can be used to inform screening policies for CRC or other diseases. For CRC, more debate is needed on what features a policy needs to exhibit to make it suitable for implementation in practice.

## 1. Introduction

Colorectal cancer (CRC) is an important cause of cancer deaths. In 2020, it was the third most incident cancer type and the second leading cause of cancer deaths worldwide (Sung et al., 2021). CRC is preventable through screening, and screening programs for CRC have been implemented in many countries. A large proportion of these are based on the Fecal Immunochemical Test (FIT) (Schreuders et al., 2015). This test measures the concentration of hemoglobin (Hb) in an individual's stool sample. An increased concentration may be caused by a precancerous lesion or a cancer. Participants with a concentration above a prespecified threshold for a positive result, commonly referred to as the cutoff, are referred for a colonoscopy, an endoscopic test with which the colon and rectum are directly observed by a specialized practitioner. Participants with a concentration below the cutoff are invited for a new FIT after a fixed time interval.

However, the FIT provides opportunities which currently remain unexploited. Grobbee et al. (2017) showed that measured FIT-concentrations, also below the cutoff, are strongly associated with the future risk of developing CRC. While screening intervals and cutoffs are equal across the population in current FIT-based programs, Grobbee et al. (2017) conclude that FIT-programs may be improved by implementing a screening policy with personalized intervals and cutoffs based on an individual's history of measured fecal Hb-concentrations.

Screening policies come with benefits, as they are likely to prevent CRC cases, and with harms such as overtreatment, for example when participants are treated for screen-detected lesions that would not have progressed to a cancer during their lifetime. These harms and benefits are measured in Quality-Adjusted Life Years (QALYs): one QALY represents one life year in perfect health. Screening policies also come with costs. Given their financial budget, policy makers aim to maximize the number of QALYs gained, and screening policies need to be developed that achieve precisely this. Implementing personalized screening policies may help to achieve this.

The amount of feasible personalized screening policies is endless, making it infeasible to evaluate the costs and health benefits of all of them in practice in randomized controlled trials. Instead, advanced simulation models such as those by Loeve et al. (1999) and Rutter and Savarino (2010) have been developed to evaluate screening policies. Still, the sheer amount of possible personalized screening options based on FIT-concentrations is so large, that it prohibits evaluating all options even by simulation. This underlines the need for optimization algorithms to design effective personalized screening policies without the need to evaluate all options.

Though algorithms have been developed to optimize personalized policies, none of them have the flexibility to incorporate detailed and computationally heavy simulation models, which is required for accurate evaluation of costs and benefits. Instead, strong assumptions are typically imposed to ensure computational tractability. Maillart et al. (2008); Ayer et al. (2012); Erenay et al. (2014) and Otten et al. (2017) use the framework of (Partially Observable) Markov Decision Processes (POMDPs) to develop personalized screening policies for a variety of cancer types, modeling the progression of the cancer by a Markov process. However, these Markov models assume, for example, that the transition rates between the different cancer states are independent. In reality, these transition rates are highly correlated within an individual. Consequently, POMDPs optimize their policies to a simpler model of the disease progression. Ahuja et al. (2017) adapt a method for POMDPs to incorporate such correlations in the cancer progression. However, they impose strong restrictions to the costs associated with screening and treatment, and don't allow for optimizing the costs and benefits as a bi-objective problem.

In this study, we present an algorithm that optimizes screening policies while incorporating MISCAN-Colon (Loeve et al., 1999). This is a detailed simulation model for CRC screening which is able to realistically evaluate the costs of and QALYs gained by a screening policy and that is commonly used to inform e.g., the United States Preventive Services Task Force on their CRC screening policy (Knudsen et al., 2020). We present a bi-objective evolutionary algorithm (EA), a heuristic algorithm which is frequently applied to difficult optimization problems. An EA is an ideal tool to combine with a computationally heavy evaluation procedure, in this case required to evaluate the costs and QALYs of a screening policy with MISCAN-Colon. Moreover, the EA is very well-suited to generate a frontier of screening policies with varying preference weights for costs and benefits, allowing policy makers to make a well-informed choice for a particular screening policy within their given budget. Finally, the EA is a flexible tool that is to some extent modular for the evaluation procedure. This means that the algorithm can be applied to inform screening programs for any disease, as long as there is a simulation tool to evaluate the costs and benefits of a screening policy, and the program uses a test with a quantitative test result. Examples include prostate cancer screening based on Prostate Specific Antigen (PSA), lung cancer screening based on smoking behavior and breast cancer screening based on nodule size, for all of which model consortia exist within the Cancer Intervention and Surveillance Modeling Network (CISNET) (Gulati et al., 2011; Alagoz et al., 2018; Criss et al., 2019).

In this paper, we present a proof-of-concept of our computational approach by (1) showing how our evolutionary algorithm can be combined with an established simulation model to optimize personalized screening policies, and (2) showing the potential of personalized screening in the case of CRC.

The remainder of this paper is structured as follows. In section 2, we discuss all aspects of the algorithm and how personalized screening policies are evaluated. In section 3, we present the outcomes of our experiments and compare them with screening policies from practice. Finally, in section 4 we discuss the outcomes of the experiments and the advantages and limitations of our algorithm.

## 2. Methods

In this section, we introduce all aspects related to our evolutionary algorithm and the experiments we performed. First, we give background on the microsimulation model MISCAN-Colon that is used to evaluate the costs and benefits of personalized screening policies obtained by the algorithm. Next, we introduce our mathematical framework for personalized screening policies. After that, we formalize the bi-objective optimization problem that we aim to solve in this study using our algorithm. Then, we present all details on the evolutionary algorithm. Finally, we introduce the experiments that we used to illustrate the performance of the algorithm.

### 2.1. MISCAN-Colon

The microsimulation model MISCAN-Colon was developed by the Department of Public Health within Erasmus University Medical Center, Rotterdam, The Netherlands. It is an established model, and has been used to inform the American Cancer Society (ACS) and the United States Preventive Services Task Force (USPSTF) guidelines (Knudsen et al., 2020). It has been validated on the results of three clinical trials on the effects of screening for colorectal cancer: the United Kingdom Flexible Sigmoidoscopy Screening (UKFSS) trial (DeYoreo et al., 2020); the Norwegian Colorectal Cancer Prevention (NORCCAP) trial (Buskermolen et al., 2018); and the Screening for Colon and Rectum (SCORE) trial (Gini et al., 2021).

The structure of the model, the underlying assumptions, and the calibration and validation studies have been described in detail by Loeve et al. (1999) and van Hees et al. (2014). In brief, the model simulates individual life histories from birth to death. At birth, all individuals are free of disease, but they may develop CRC during their lives. MISCAN-Colon assumes that all cancers develop from precancerous lesions, called adenomas, via the conventional adenoma-carcinoma pathway. Individuals may develop one or more adenomas over time. These lesions grow and may progress to preclinical CRC. Preclinical cancers are asymptomatic but may become symptomatic, resulting in clinical detection. Once a cancer becomes clinical, the person is treated, and a time to death is determined, depending on the stage of the cancer. The parameters of the natural history of CRC were calibrated to high-quality data sources, such as autopsy studies on age-specific adenoma prevalence and multiplicity (Meester et al., 2018) and age-, stage-, and location-specific CRC incidence data from the Surveillance, Epidemiology and End Results (SEER) program from the period before screening was common practice (1975–1979) (SEER, 2021).

The model also has an optional screening component. When activated, the simulated individuals undergo screening according to a specified screening policy. Some lifetimes are altered because some cancers are prevented by removal of the precedent adenomas, or are detected at an early stage, leading to more favorable survival. The effect of screening depends on the implemented policy and the test characteristics such as the sensitivity and specificity and the reach of endoscopic tests. Endoscopic tests also have a risk of complications. The characteristics of the screening tests in MISCAN-Colon are based on various studies to assess the diagnostic performance of FIT and colonoscopy (Knudsen et al., 2016).

Screening policies are associated with monetary costs and benefits in terms of QALYs, related to the total number of screening tests and the life years spent on cancer treatment in a simulated population. After simulation, the model aggregates these quantities to calculate the policy's costs and benefits. The costs and benefits used in this study are listed in Gini et al. (2017).

Up to now, MISCAN-Colon has not been used before to evaluate personalized screening policies based on FIT-history. FITs were modeled as binary tests that return either a positive or negative test result based on sensitivity and specificity. For our study, the model was extended with a prototype module describing individuals' fecal occult blood loss over time, such that FIT-concentrations were returned. A model was developed with a linear mixed-effects model (GLMM) structure. Its parameter values were estimated using population-based data on measured FIT-concentrations and corresponding outcomes observed in the Dutch national colorectal cancer screening program (Toes-Zoutendijk et al., 2017). This module is a prototype and still needs further calibration before informing actual policy changes. However, the quality of this module is not relevant for the purpose of this study which is to provide a proof-of-concept of the presented computational technique.

An overview of the model assumptions for the natural history, test characteristics and the module for FIT-concentrations is presented in Supplementary Section 1 of the Supplementary Material.

### 2.2. Personalized Screening Policies

**Figure 1:**
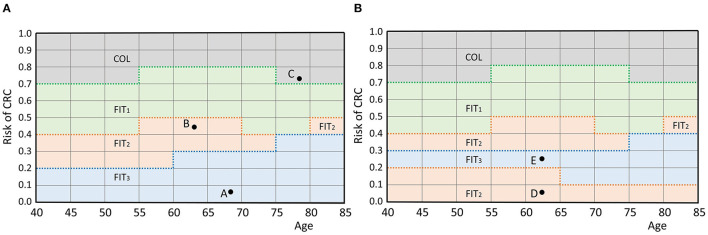
**(A)** An example policy. Individuals are represented by a pair (age, perceived risk) which is a point in the belief space. For example, the participant represented by A is aged 68 and has a risk of 0.075. A policy defines an action for each individual by partitioning the belief space and relating each part to an action, as shown by the colors. The individuals represented by points A and B are prescribed actions *FIT*_3_ and *FIT*_2_ (a screening interval of 3 and 2 years), respectively. Individual C is referred to a hospital for a colonoscopy. The discretization of R and T causes the grid structure. **(B)** An infeasible policy. Participant E is assigned a longer interval than participant D while they are of the same age and the risk of E is higher. Therefore, this policy does not comply with the test order assumption.

In this section, we provide the mathematical framework for personalized screening policies. In short, an individual is represented by a pair (*r*, τ) that contains its perceived risk of CRC based on its FIT-history *r* and its age at the most recent FIT τ. The two-dimensional space of all possible pairs is called the *belief space*. Each individual is represented by a point in this space. A screening policy prescribes an action for each point in the belief space. There are two types of actions: either a participant is referred to a hospital for a follow-up colonoscopy, denoted by *COL*, or an interval of *I* years until the next FIT is prescribed, denoted by *FIT*_*I*_. In fact, a personalized policy is a mapping that partitions the belief space and relates each part to an action. An example is given in Figure 1A, in which screening intervals of 1, 2 and 3 years are prescribed. The remainder of this section provides a more extensive formalization of the framework of personalized screening policies.

First, the framework requires a discrete set of screen-eligible age groups T. In this study, individuals were assumed eligible for screening between ages 40 and 85. This range was split in age groups of 5 years and we assumed that the policy is the same for each age group, i.e., two individuals aged 40 and 44 with equal perceived risk are prescribed the same action. Age groups are represented by their lowest age and in our study, the set of age groups was T:={40,45,…,80}.

Second, the framework requires a measure for perceived risk of CRC. In this study, perceived risk was estimated by the average of an individual's *k* most recently measured FIT-concentrations. We used *k* = 1 as a base case and *k* = 2, 3 for sensitivity analyses. The average was mapped linearly to a value in the range [0, 1] where risk values of 0 corresponded to a negligible risk and 1 to a very high risk. This way, more advanced risk estimators can easily be incorporated in the future. Since most countries use a cutoff between 15 and 80 μg/g (Schreuders et al., 2015), we assumed that average FIT-concentrations above 100 μg/g correspond with a perceived risk of 1. In our method, individuals with a FIT-concentration above 100 μg/g were always referred for a colonoscopy. Formalizing the above, the perceived risk of CRC *R*^*k*^ after the participant's *n*^*th*^ FIT was calculated as


$$\begin{array}{r} R^{k}:=\frac{1}{100k}\overset{k}{\underset{i=0}{\sum }}C_{n-i}, \end{array}$$

with *C*_*j*_ the measured concentration at the participant's *j*^*th*^ FIT. Similar to the age groups, we discretized the interval [0,1] in parts of length 0.1 and assumed that the action is the same within each part for a given age group, i.e., two individuals with risk 0.11 and 0.19 of equal age were prescribed the same action. This restricted the number of feasible cutoffs. The set of feasible cutoffs R was {0, 0.1, …, 1}. Note that the discrete nature of T and R causes the grid structure in Figure 1A. Finer discretization increases the number of potential personalized screening policies, but also increases the size of the search space of the algorithm.

Third, the framework needs a set of actions A. In our study, we used two types of actions. The first, denoted by *COL*, was equivalent to a positive FIT and referred an individual for a colonoscopy in a hospital. After a positive colonoscopy result, the individual left the screening program and was referred to a surveillance program instead. After a negative colonoscopy result, the individual re-entered the screening program and obtained a new FIT after a fixed 5-year interval. The second action type corresponded with a negative FIT and prescribed a screening interval *I*. Such actions were denoted by *FIT*_*I*_. The set of feasible intervals was denoted by I. The resulting action set A was


$$\begin{array}{r} A:=\left\{COL\right\}\cup \left\{FITI|I\in I\right\}. \end{array}$$

Considering larger action sets allows for more potential screening policies, but also increases the size of the algorithm's search space.

The space 𝔹:=R×T is called the *belief space*. The current status of a participant is represented by a point in this space. A screening policy π:𝔹→A partitions the belief space and maps each part to an action in the action set (see Figure 1A), defining an action for each participant.

The framework assumes that the actions have a test burden and that the order of the actions in the belief space is fixed with respect to this test burden. In our case, colonoscopies, for which participants are referred to a hospital, have a relatively high test burden compared to FIT which is done at home. Short FIT-intervals were also assumed to have a higher burden than longer intervals. Only screening policies that adhere to this ordering by test burden are considered. That is, a participant is only assigned a test with a higher burden than another participant of the same age, if also the perceived risk is higher. Figure 1B shows an example of a screening policy that does not comply with the *test order assumption*. We consider such a policy infeasible in this framework.

As the ordering of the actions is fixed per age group, screening policies can also be characterized by the bounds of their partitions. The upper bound of the parts of the belief space that correspond to an action are considered a function in the belief space. In our study, this concerned the actions *FIT*_*I*_ with corresponding policy bounds βI:T→R. In Figure 1A, these functions are represented by the bold, dotted lines. A screening policy is characterized by the set of its policy bounds


$$\begin{array}{r} \pi =\left\{\beta I\right\}I\in I. \end{array}$$

Note that the characterization only included the policy bounds of the screening intervals I∈I, because the upper bound of the part corresponding with the action *COL* was not relevant. This characterization of personalized screening policies is used in the remainder of this paper.

Policies that are obtained by a combination of two other policies are also considered. By prescribing policy π to a fraction λ ∈ (0, 1) of the population and prescribing policy σ to the remaining fraction (1−λ), a new policy ρ is generated.

### 2.3. Optimization Problem

Next, we introduce the optimization problem solved in this paper. In particular we present a multi-objective (specifically bi-objective) optimization problem.

A policy π has associated costs and QALYs, denoted as *C*(π) and *Q*(π), respectively, and measured per 1,000 individuals, as is common. We define $o\left(\pi \right):=\left[\begin{array}{c} C\left(\pi \right)\\ Q\left(\pi \right) \end{array}\right]$as the vector containing both objectives of π.

The bi-objective optimization problem is to find policies minimizing the costs and maximizing the QALYs gained. A single policy optimizing both objectives is unlikely to exist as screening policies with an increased number of QALYs gained generally come with higher costs. Therefore, we aim to find a set of policies that contains those with maximal benefits for given costs. Given this set, policy makers can choose policies based on their budget constraints or on what they find a suitable balance between the two criteria.

In a multi-objective setting, the concept of Pareto dominance is used to compare policies. A policy π *dominates* another policy σ if π is a better choice than σ, i.e., if (1) the costs and QALYs of π are at least as good as those of σ:


$$\begin{array}{r} Q\left(\pi \right)\geq Q\left(\sigma \right)\text{ and }C\left(\pi \right)\leq C\left(\sigma \right), \end{array}$$

and (2) at least one of the objectives is better:


$$\begin{array}{r} Q\left(\pi \right)>Q\left(\sigma \right)\text{ or }C\left(\pi \right)<C\left(\sigma \right). \end{array}$$

**Figure 2:**
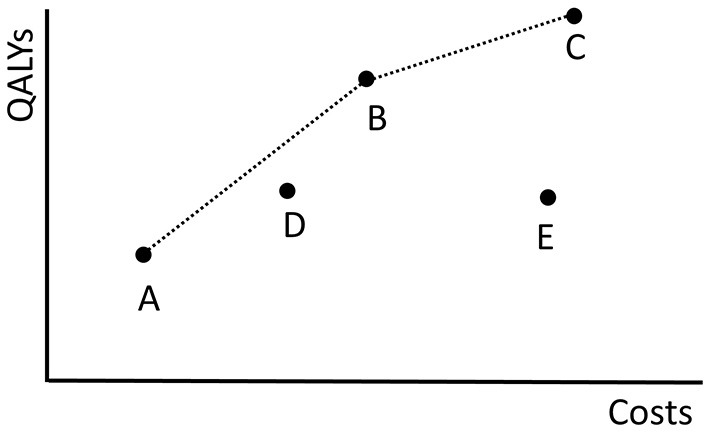
The costs of and QALYs gained by the fictive screening policies in Π = {*A, B, C, D, E*} are plotted. The dotted black line shows the objective values of the policies in the approximation set ψ(Π). The minimal representation of ψ(Π) is {*A, B, C*}.

Figure 2 shows the costs and QALYs of several example policies. Here, policy *B* dominates *E* because its costs are lower and its QALYs are higher. *B* does not dominate *D*. A policy that is not dominated by any other policy is called *Pareto optimal*. The set of all Pareto optimal policies is referred to as the *Pareto frontier*. The multi-objective optimization problem is to find the Pareto frontier. The Pareto frontier potentially includes an infinite number of policies, and is computationally difficult to identify precisely. Therefore, the algorithm aims to find a set of policies that best approximates the Pareto frontier.

Next, we explain how an approximation of the Pareto frontier is represented using the approximation set as introduced in Zitzler et al. (2003). This set makes use of combinations of policies, i.e., prescribing policy π to a fraction λ ∈ (0, 1) of the population and prescribing policy σ to the remaining fraction (1−λ) which results in a new policy ρ. Observe that the objective values of ρ are convex combinations of the objective values of π and σ in the conventional sense:


$$\begin{array}{r} o\left(\rho \right)=\lambda o\left(\pi \right)+\left(1-\lambda \right)o\left(\sigma \right). \end{array}$$

By varying λ, an infinite number of new policies can be generated using only two policies.

We use the above observation to create an approximation set of the Pareto frontier of the following form. An approximation set is represented using a finite set of policies Π. This approximation set contains all non-dominated policies among Π and all their non-dominated combinations, and is denoted by ψ(Π). This way, (if |Π| ≥ 2) the approximation ψ(Π) consists of an infinite set of policies, but can be represented using a, typically small, finite set of policies. In our computations, but also when presenting the results in this paper, it is beneficial to consider a minimal representation of ψ(Π), which is a smallest subset Π′ of Π such that ψ(Π′) = ψ(Π).

As an example, the dotted black line in Figure 2 shows the approximation set ψ(Π) represented by Π = {*A, B, C, D, E*}. The same approximation set can also be represented by policies Π′ = {*A, B, C*} because *D* is dominated by a combination of *A* and *B* and *E* is dominated by *B*.

### 2.4. Evolutionary Algorithm

In this section, we describe the evolutionary algorithm (EA) which we developed to identify approximation sets of the Pareto frontier. EAs are based on the principle of *survival of the fittest* (Holland, 1975).

In general, the algorithm keeps track of two sets of policies. Firstly, it maintains a *population* of screening policies. This set evolves over time, i.e., it changes at every iteration of the EA. Secondly, it maintains a *memory* which is a set of policies that is a minimal representation of the best approximation set found so far. This set is updated every time that a policy appears in the population which is non-dominated by any found policy. This new policy is then added to the memory, and others are removed if they are dominated. Therefore, the population can be thought of as the current generation, while the memory simply contains the best policies observed over all generations. Although we are interested in the approximation set represented by the final memory as the final solution to our optimization problem, the population does not necessarily have to be a non-dominated set of policies. In fact, for diversification purposes it can be beneficial to allow inferior policies in the population.

As an example, if policies *A*, …, *E* in Figure 2 are the policies found by the algorithm, policies *A*, …, *D* form the memory as policy *E* is dominated by *B*. Policy *D* is also part of the memory because it is not dominated by a found policy.

The EA starts with an *initial population* that consists of a predefined number of screening policies. It evaluates the *fitness*, or quality, of these policies in terms of the objectives. Then, it *selects* half of the policies to stay in the population and discards the other half. This is a semi-random selection procedure where solutions of higher fitness are more likely to be selected. The selected policies are paired up randomly to form pairs of parents. Together, these parents generate two child policies by exchanging some of their features, called *cross-over*. Some of the child policies undergo random *mutations* in which their features are changed randomly. Finally, the algorithm adds the children to the population, which results in a new population, and updates the memory such that it contains the best policies observed until then. It repeats the cycle of fitness evaluation, selection, cross-over and mutation until some stopping criterion is met.

In the remainder of this section we provide a more detailed description of the key elements of the EA and its interaction with MISCAN-Colon.

#### 2.4.1. Initialization

**Figure 3:**
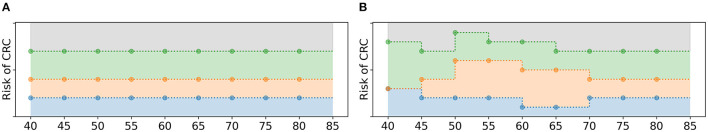
Schematic overview of the initialization of a policy. **(A)** First, the policy bounds are assigned a constant value over all age groups, adhering to the test order assumption. **(B)** After that, random mutations are applied.

A screening policy is initialized in two steps as illustrated in Figure 3. First, each policy bound β*I*(τ) is assigned a constant value for all age groups τ∈T. For that, |I| random values are uniformly drawn from R and assigned to the policy bounds, adhering to the test order assumption. That is, the smallest value drawn from R is assigned to the policy bound that relates to the action with the lowest test burden, the second smallest value to the action with the second lowest test burden, etc. Then, the mutation operator as described in section 2.4.5 is applied such that the policy bounds are not necessarily constant over the age groups anymore. The algorithm repeats these two steps *N*_*pop*_ times to obtain an initial population of policies, where *N*_*pop*_ denotes the number of screening policies in the population.

#### 2.4.2. Fitness Evaluation

The algorithm bases the fitness of a policy in the population on its costs and QALYs as simulated by MISCAN-Colon. In MISCAN-Colon, both objectives were discounted by 3% annually from the age of 40 and were calculated relative to a situation without screening. Simulations used one million individuals. Common seeds ensured that the results of different simulation runs were comparable.

**Figure 4:**
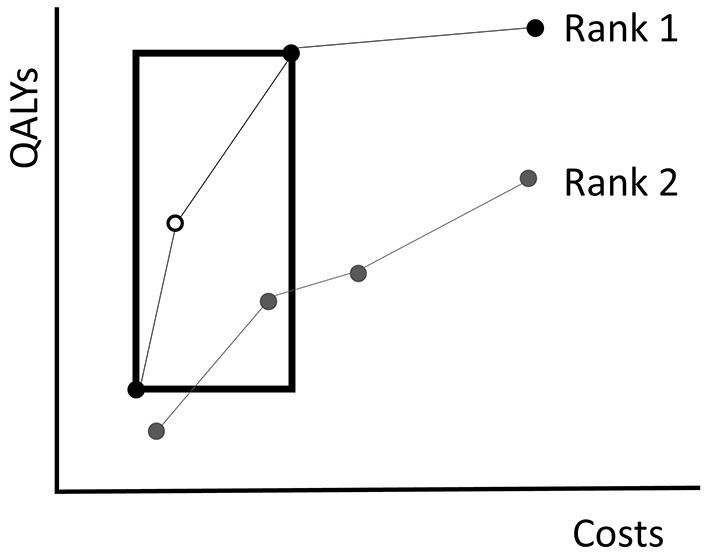
An illustration of the NSGA-II algorithm. The dots represent the costs of and QALYs gained by several fictive policies. Policies with equal rank are connected by the lines. Non-dominated policies obtain rank 1. Those that are dominated by policies with rank 1 only, obtain rank 2, etc. The crowding distance of the white policy equals the circumference of the rectangle drawn around it. The rectangle touches the next less costly and next more expensive policies with equal rank.

The EA uses the Non-Dominated Sorting Genetic Algorithm-II (NSGA-II) introduced by Deb et al. (2002) to evaluate fitness. NSGA-II summarizes fitness of policies with two quantities: the *rank* and *crowding distance*. Given a population of policies *P*, the rank of a policy represents to what extent it is dominated by other policies in *P* (excluding combinations of policies). Non-dominated policies in *P* obtain rank 1. Then these policies are excluded and the non-dominated policies of the remainder are assigned rank 2. This is repeated until every policy is ranked (Figure 4). Consequently, the solution quality increases for decreasing rank.

It is likely that multiple policies in the population obtain an equal rank. To break a tie in the selection procedure, NSGA-II evaluates for each policy a crowding distance. The crowding distance is a statistic that reflects the level of isolation with respect to other policies with equal rank. For a policy π, the crowding distance is the circumference of the rectangle that touches the next less costly and next more expensive policies with the same rank as π, see Figure 4 for an example. Note that the crowding distance of the cheapest and most expensive policies in a frontier are considered to be infinite. In case two policies have equal rank, the algorithm prefers the one with a higher crowding distance. The idea behind the crowding distance is to get a good spread of different screening policies, i.e., expensive as well as cheap policies. This may help in achieving a high quality approximation of the complete Pareto frontier.

In our particular bi-objective case, the time complexity of the NSGA-II algorithm is $O\left(N_{pop}^{2}\right)$ (Deb et al., 2002).

#### 2.4.3. Selection Operator

Once the rank and the crowding distance of the policies in the population are evaluated, the EA selects which policies are maintained in the population and which are discarded. The maintained policies form the *mating pool*. In iteration *g*, the pool is denoted by *M*_*g*_. During the selection procedure, exactly *N*_*sel*_ := *N*_*pop*_/2 policies are selected and added to *M*_*g*_. The selection operator consists of two phases.

First, the mating pool is (partially) filled by an elitist selection procedure. Given the current population *P*_*g*_ and the approximation set ψ(*P*_*g*_), the EA adds the solutions in the minimal representation of ψ(*P*_*g*_) to the mating pool, i.e., it adds the policies in the population that are not dominated by (combinations of) other policies in the population. This ensures that the best policies are selected. Note that this is a subset of the policies with rank 1. Tests with our benchmark have shown that adding the complete set of policies with rank 1 in this phase leads to poorer algorithm performance. If more than *N*_*sel*_ policies are selected in this first, elitist phase, the algorithm randomly discards policies until *N*_*sel*_ policies remain.

In the second phase, the remainder of the mating pool is filled by tournament selection: two policies are randomly sampled from the population and the fittest of the two policies in terms of rank and crowding distance is added to the mating pool. This is repeated until the mating pool is filled with *N*_*sel*_ policies. Note that this procedure may lead to duplicates in the mating pool. Policies may be selected once in both phases and/or multiple times in the second phase.

#### 2.4.4. Cross-Over Operator

**Figure 5:**
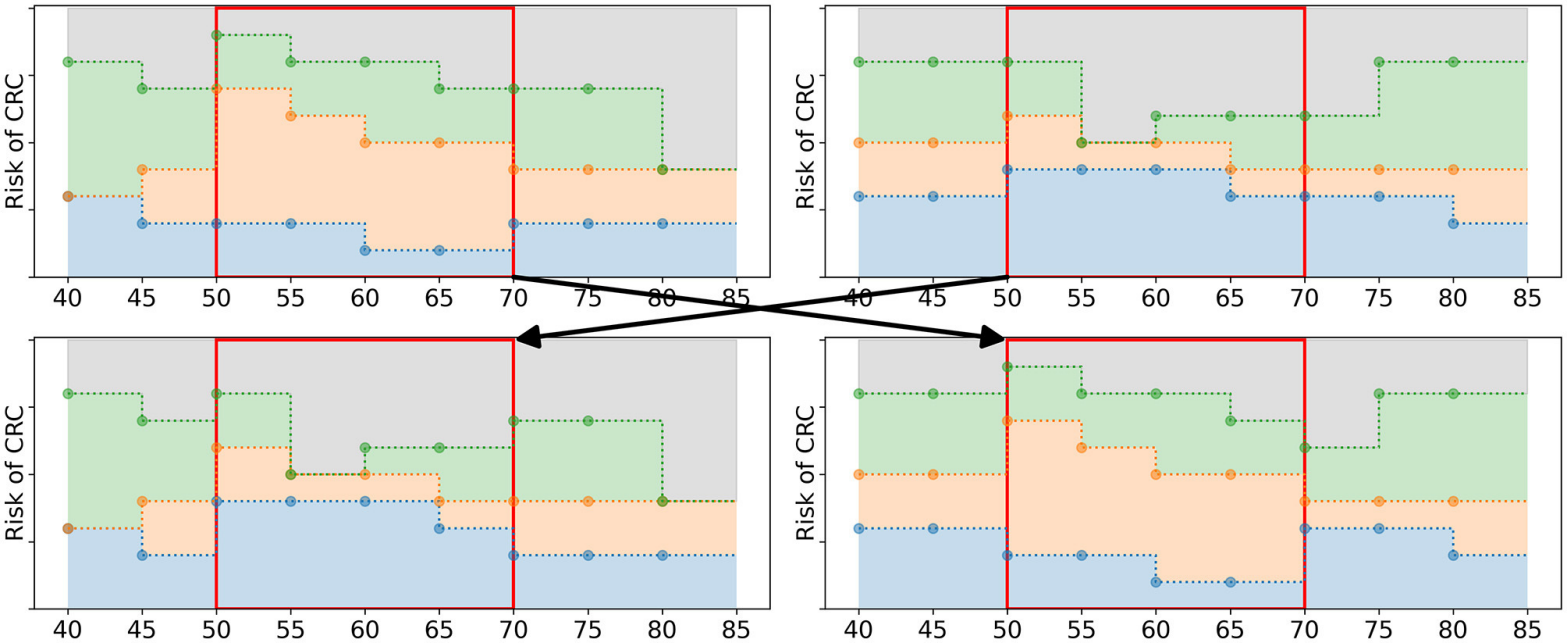
Schematic overview of the cross-over operator. The two upper figures represent the parent policies, a random pair of policies in *M*_*g*_. The lower figures represent two offspring policies that are added to *O*_*g*_. The two age groups 50 and 65 were randomly selected. The policy bounds in between these age groups (marked by the red box) are exchanged in the offspring.

Having filled the mating pool *M*_*g*_, the algorithm applies 2-point cross-over (Whitley, 1994) to generate offspring. The policies in *M*_*g*_ are paired up randomly. For each of the pairs, two age groups $\tau_{1},\tau_{2}\in T$ are randomly selected. The policy bounds in the interval [τ_1_, τ_2_] are exchanged, see Figure 5 for an example. This results in two new offspring policies which are added to *O*_*g*_, the set of offspring obtained in iteration *g*. After all pairs of parents have generated offspring, *O*_*g*_ has a size of *N*_*pop*_/2.

#### 2.4.5. Mutation Operator

**Figure 6:**
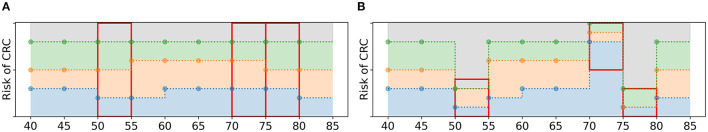
Schematic overview of the mutation of a policy. **(A)** A policy before mutation. If *p*_*e*_ = 3/9, the algorithm is likely to select three age groups, marked by the three red boxes. **(B)** For each age group, new policy bounds are sampled from a subset of R (red boxes). For age groups 50 and 75, $\tilde{r}$ is 0.4 and 0.3, resp., and it is an upper bound. Therefore, the new values of the policy bounds must be smaller or equal to 0.4 and 0.3, resp. For age group 70, $\tilde{r}=0.5$ is a lower bound and all new values are larger or equal to 0.5.

The offspring policies in *O*_*g*_ are subject to random mutations with probability *p*_*M*_. If the EA selects a policy to undergo mutation, the following steps are taken. First, a fraction *p*_*e*_ of the age groups in T is randomly selected. For these age groups, the values of all policy bounds {βI}I∈I are mutated: they are replaced by random values from R. However, these values are not sampled from R, instead they are sampled from a subset of R. For each selected age group, a value $\tilde{r}\in R$ is sampled. This value is an upper or a lower bound with 50% probability. If it is an upper bound, |I| random values are drawn from the values in R smaller or equal to $\tilde{r}$. If it is a lower bound, they are drawn from the values in R larger or equal to $\tilde{r}$. These new values are assigned as policy bounds, adhering to the test order assumption, see Figure 6 for an example.

The reason to sample the new values from a subset of R is that this is more likely to result in a larger variety of policies. For example, the policy bound related to the largest screening interval always obtains the smallest of the |I| new values. If these values are drawn from the complete set R, it is unlikely that a value close to 1 is assigned to this bound. This is more likely to occur when sampling from a subinterval of R.

#### 2.4.6. Updating Procedures and Stopping Condition

After applying all operators, the algorithm obtains (1) a mating pool *M*_*g*_ that contains the selected policies from the current population *P*_*g*_, and (2) a set of newly generated offspring *O*_*g*_. Both sets have size *N*_*pop*_/2. The algorithm merges these sets to obtain the population for the next iteration, i.e., *P*_*g*+1_ = *M*_*g*_ ∪ *O*_*g*_.

Additionally, it updates its memory with the best found policies. It adds all newly found policies that are not dominated by the policies in the current memory, and removes all policies that are dominated by the newly added policies.

The algorithm repeats the procedures for selection, fitness, cross-over, mutation and updating until no new solutions are added to the memory for *N*_*stop*_ = 30 consecutive iterations. The approximation set represented by the memory at the final iteration is considered the best approximation of the Pareto frontier and is the final solution to our problem.

### 2.5. Experiments and Implementation

We demonstrate the performance of the algorithm with three different experiments. First, we evaluated how well the algorithm approximated a Pareto frontier, i.e., the optimal solution to the multi-objective optimization problem, using a benchmark problem. We considered an instance of the problem with a relatively small number of feasible policies, which enabled us to enumerate all feasible policies, evaluate their costs and QALYs and identify the Pareto frontier. All policies were simulated with 2 million individuals using common random numbers to ensure that each policy was evaluated for exactly the same population. Based on this benchmark, we also identified the best values for the parameters *N*_*pop*_, *p*_*M*_ and *p*_*e*_.

The benchmark problem size was reduced by restricting the assumed screen eligibility to ages 55 to 75, resulting in the age groups T={55,60,65,70}, and restricting the set of feasible cutoffs to R={0,0.125,0.25,0.375,0.5}. We used *R*^1^ to estimate perceived risk. As shown in Supplementary Section 2 of the Supplementary Material, this combination of parameters gave approximately 1.5 million feasible policies.

**Figure 7:**
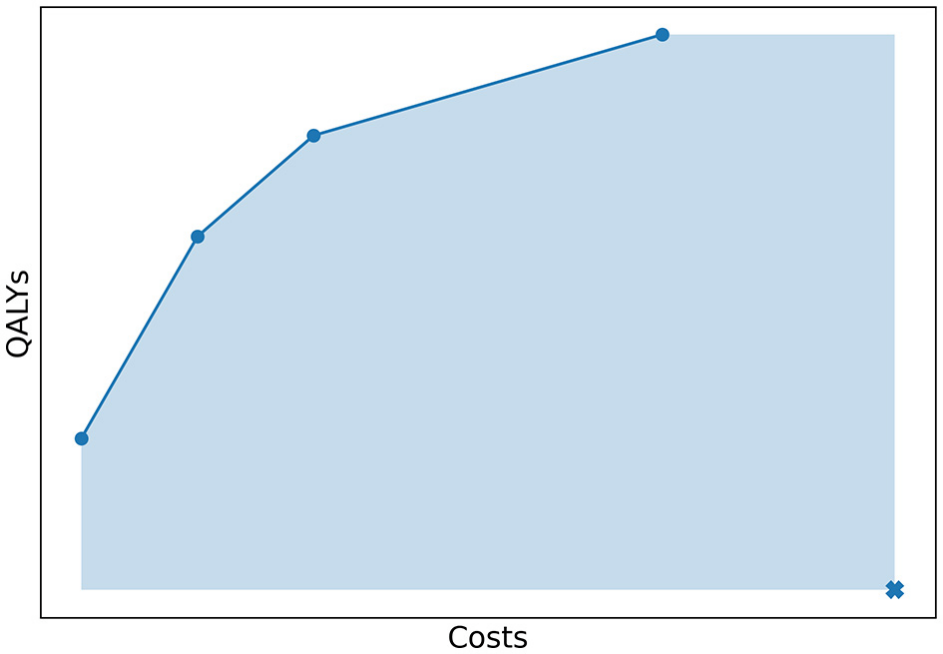
The costs and QALYs of several policies that form a minimal representation of an approximation set. The hypervolume of this approximation set is equal to the area of the objective space dominated by the approximation set, bounded by a reference point. This point is marked with a cross in the figure.

To quantify how well the Pareto frontier was approached by an approximation set, we used the relative difference between the hypervolume (HV) of both sets. The HV is a quality indicator introduced by Zitzler and Thiele (1998) and is very common in multi-objective optimization (Riquelme et al., 2015). In our study, the hypervolume of an approximation set was defined as the area of the objective space dominated by the approximation set, bounded in some sense by a reference point as illustrated in Figure 7. The reference point was chosen as (costs, QALYs) = (4,000,000; 0). In Experiment 1, we evaluated the HV of both the approximation set represented by the Pareto frontier and the approximation set obtained by the algorithm. The relative difference between the two quantified the optimality gap, i.e., how well the approximation set approaches the Pareto frontier.

Next, we used two larger problem instances to test the algorithm. Experiment 2 used the original settings for T and R and used the action set $A=\left\{COL,FIT_{1},FIT_{2},FIT_{3}\right\}$ such that both the cutoff for FIT-positivity and screening intervals were optimized. In Experiment 3, we considered a simplified situation in which $A=\left\{COL,FIT_{2}\right\}$. It effectively means that we used a fixed screening interval of 2 years and only optimized the cutoff per age group. This is an improvement already compared to current practice in which the cutoff is fixed for all ages. The size of the search space was much smaller compared to Experiment 2 (see Supplementary Material).

For both experiments, it was computationally impossible to evaluate all feasible policies and to find the exact Pareto frontier. To evaluate the obtained approximation sets, we compared them in terms of costs and QALYs with policies recently evaluated for the United States Preventive Services Task Force (USPSTF) by Knudsen et al. (2020) that include FIT and/or colonoscopies. For a fair comparison, we only used reference policies that start screening no later than age 45, because the policies generated by the algorithm all start at age 40 due to our chosen parameter settings. An overview of the reference policies is shown in Supplementary Section 3 of the Supplementary Material. The reference policies and those in the memory of the algorithm were (re-)evaluated with MISCAN-Colon using 2.5 million individuals and with a different random number stream than used in the algorithm. This prevented a biased comparison, since the policies of the algorithm may have been optimized to the random number stream used for simulations in the EA.

As is common in health economics, we made use of a statistic, the *incremental cost-effectiveness ratio* (ICER) (Sanders et al., 2016), to identify a single policy in an approximation set which is cost-effective, for comparative purposes. We evaluated the ICER for the policies in the finite set that is a minimal representation of the approximation set. The ICER of policy π is defined as the extra costs per extra QALY gained when opting for policy π instead of the next less costly policy in the minimal representation, i.e., it is defined as the ratio between the difference in costs and the difference in QALYs gained between the two. Due to our definition of an approximation set, the ICER of a policy increases for increasing costs. The cost-effective policy is defined as the policy that has maximum benefits for which the ICER is still below a predetermined threshold, often called the willingness-to-pay threshold. In this study, we used a threshold of $100,000 per QALY gained to determine the cost-effective strategy.

The running time of the algorithm strongly depends on the implementation and computational resources. In our experiments, the algorithm was implemented using the Python DEAP evolutionary computation framework (Fortin et al., 2012) and implemented as a high-performance computing (HPC) workflow using the EMEWS framework (Ozik et al., 2016). The first, second and majority of the third experiment were run on Bebop, an HPC cluster managed by the Laboratory Computing Resource Center at Argonne National Laboratory. Bebop has 1,024 nodes comprised of 672 Intel Broadwell processors with 36 cores per node and 128 GB of RAM and 372 Intel Knights Landing processors with 64 cores per node and 96 GB of RAM.

## 3. Results

In this section, we present the results of the three experiments introduced in section 2.5. All presented costs and QALYs are relative to a situation without screening for CRC. Also, they were discounted by 3% annually from age 40, as is common in cost-effectiveness analyses.

### 3.1. Experiment 1: Benchmark

**Figure 8:**
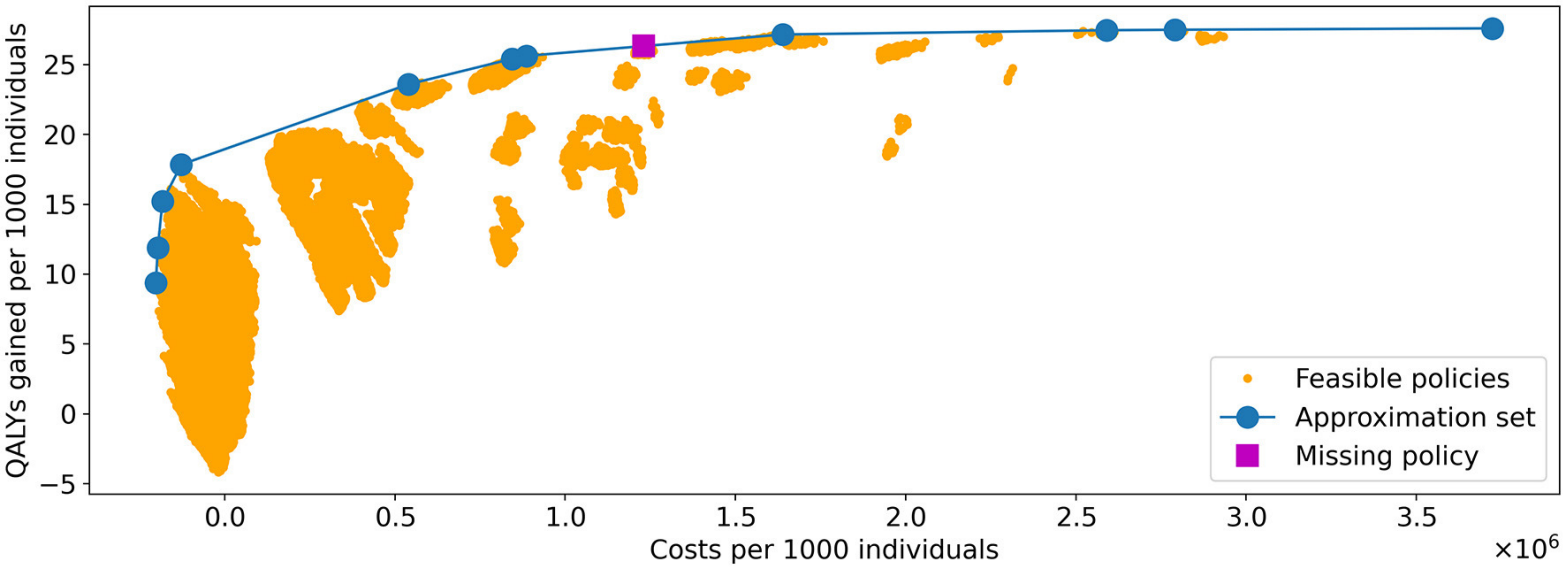
Visualization of all feasible policies in the problem instance of Experiment 1. The yellow dots represent the costs and QALYs of the feasible policies. The blue line shows the approximation set obtained by the algorithm. It is represented by the 11 policies indicated by the blue dots. The policy indicated by the purple square is the only strategy on the PF that was not in the approximation set found by the algorithm.

Figure 8 shows the costs and QALYs of all feasible policies in the benchmark problem, evaluated in 10 phases on Bebop, 9 of which used 1,792 cores each and 1 which used 2,016 cores. It was completed in 97.07 h, resulting in 177,528.31 core hours in total.

Experiments were done with varying values for *N*_*pop*_, *p*_*M*_, and *p*_*e*_. After convergence, the hypervolume (HV) of the obtained approximation set was highest for the values (*N*_*pop*_, *p*_*M*_, *p*_*e*_) = (400, 0.3, 0.6). This approximation set, obtained after 499 iterations of the algorithm, is included in Figure 8. The three selected parameters values are used in the remainder of our study.

We observe that nearly all feasible policies are dominated by the approximation set, suggesting it is a good approximation of the Pareto frontier. This is further confirmed by the hypervolume. The HV of the approximation set and Pareto frontier (PF) equal 108,116,896 and 108,124,226, respectively, effectively resulting in an optimality gap of 0.007%.

The PF contains 12 policies, the minimal representation of the approximation set contains 11. Further analysis showed that the 11 policies representing the approximation set are all part of the representation of the PF: the approximation set misses only one of the policies on the PF, which explains the optimality gap. The missing policy is marked in Figure 8.

### 3.2. Experiment 2: Optimizing Cutoffs and Screening Intervals

In the second experiment, using *R*^1^ to estimate perceived risk, the algorithm took 1263 iterations until convergence. This was performed in 5 phases on Bebop. Each phase of the experiment was run on 432 cores, enabling 430 individual policies to be evaluated in parallel with the remaining two processors being used for workflow management. The total number of 1,263 iterations was completed in 101.7 h for a total compute time of 43,934.4 core hours, four times faster than the enumeration in Experiment 1 despite the factor 10^16^ increase in search space (see Supplementary Material). The evolutionary operators consumed 0.11% of the total computation time, the remainder was used by MISCAN-Colon.

Incorporating extra FIT-concentrations in the perceived risk value did not affect the performance and the outcomes of the algorithm. Experiments with perceived risk estimators *R*^2^ and *R*^3^ resulted in similar computation times and policies with similar costs, QALYs and patterns. In the remainder of this section, we only discuss the outcomes using *R*^1^.

**Figure 9:**
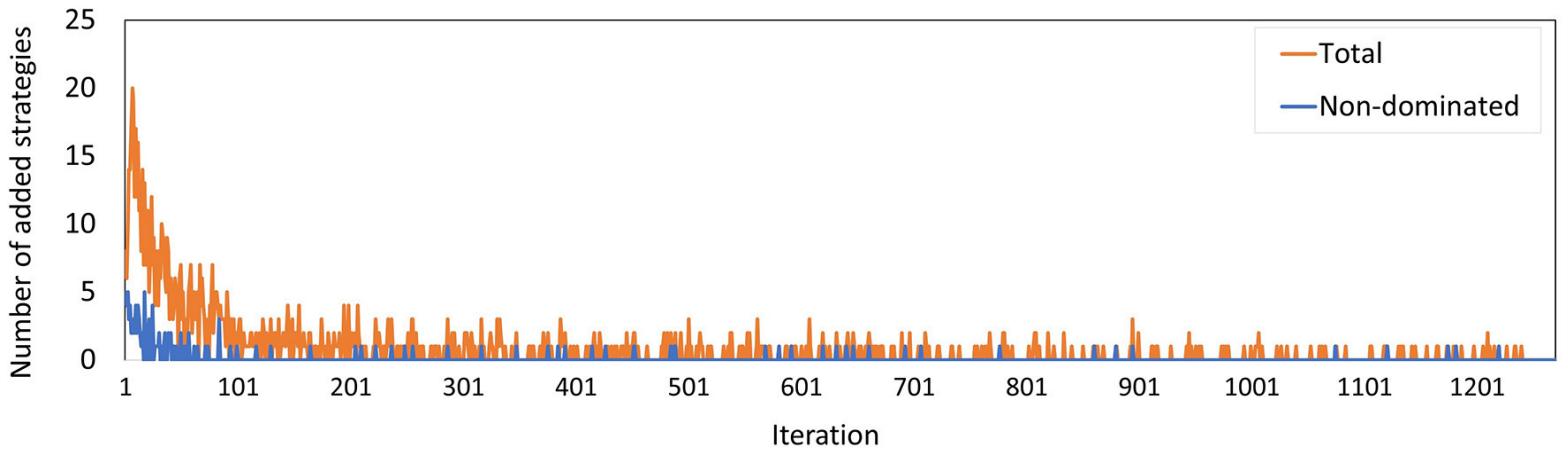
The number of policies added to the memory in each iteration for Experiment 2. The blue line counts all added policies, the orange line only those that are not dominated by (combinations of) other policies in the current memory. The latter are part of the minimal representation of the memory's approximation set.

Figure 9 shows the total number of policies added to the memory in each iteration, and how many of these policies were added to the minimal representation of its approximation set, i.e., the number of new policies that were not dominated by any *combination* of other policies in the memory. We observe that the latter group is a minority. Especially in the final 600 iterations, only 9 of such policies were found.

**Figure 10:**
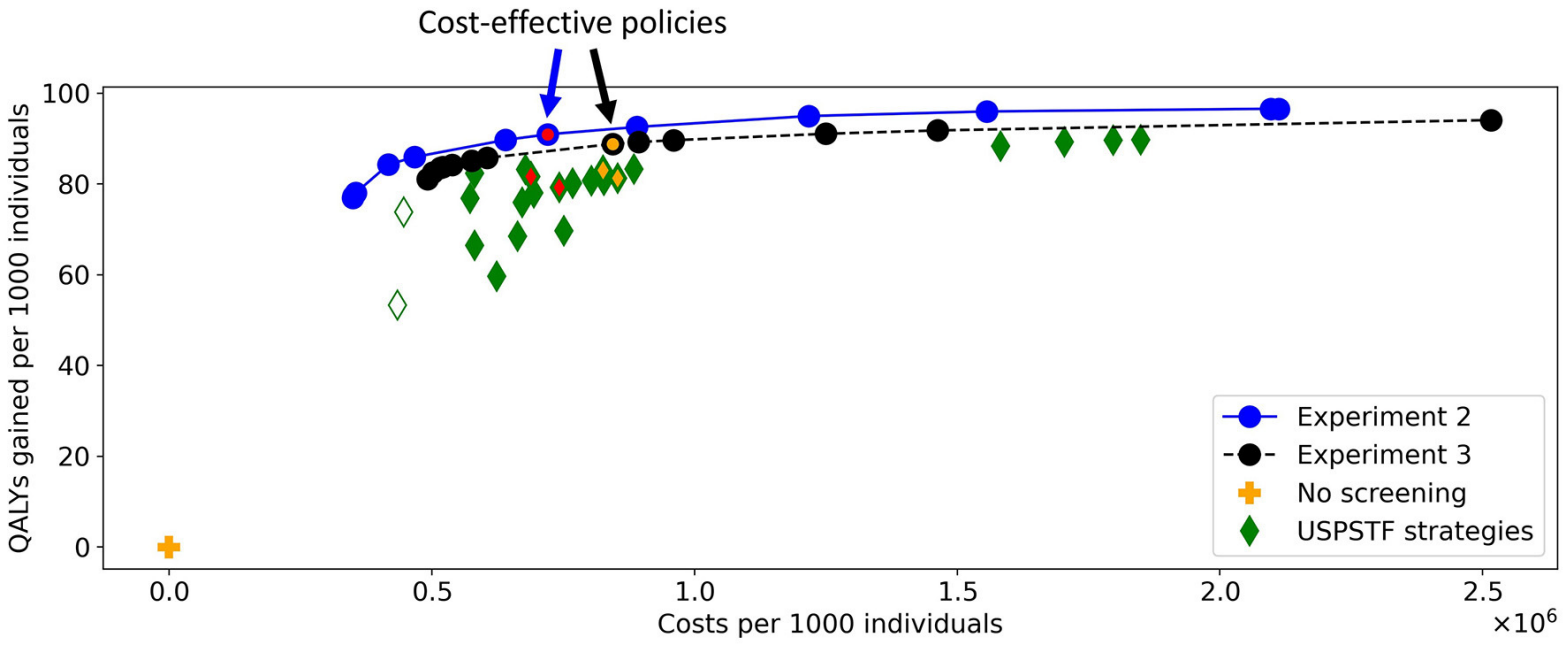
The costs and QALYs of the reference policies and of the best approximation sets generated in Experiments 2 and 3. The blue line shows the best approximation set obtained in Experiment 2, the black line shows that of Experiment 3. The blue-red and black-orange policies in the approximation sets are cost-effective. The plus represents a situation without screening, the diamonds represent the policies evaluated for the USPSTF with FIT and/or colonoscopies that start at age 45. The two white diamonds are the two reference policies that are not dominated in Experiment 3. The four green-red/green-orange diamonds are referred to in Figures 11, 14.

Figure 10 shows the costs and QALYs of the best approximation set of the PF obtained by the algorithm and of all reference policies. The minimal representation of the approximation set contains twelve personalized policies, and dominates all reference policies. For similar costs, the QALYs of the obtained screening policies increased up to 14% compared to the reference policies. This shows that the algorithm succeeded in finding personalized screening policies that are more effective than the uniform reference policies as evaluated using MISCAN-Colon.

**Figure 11:**
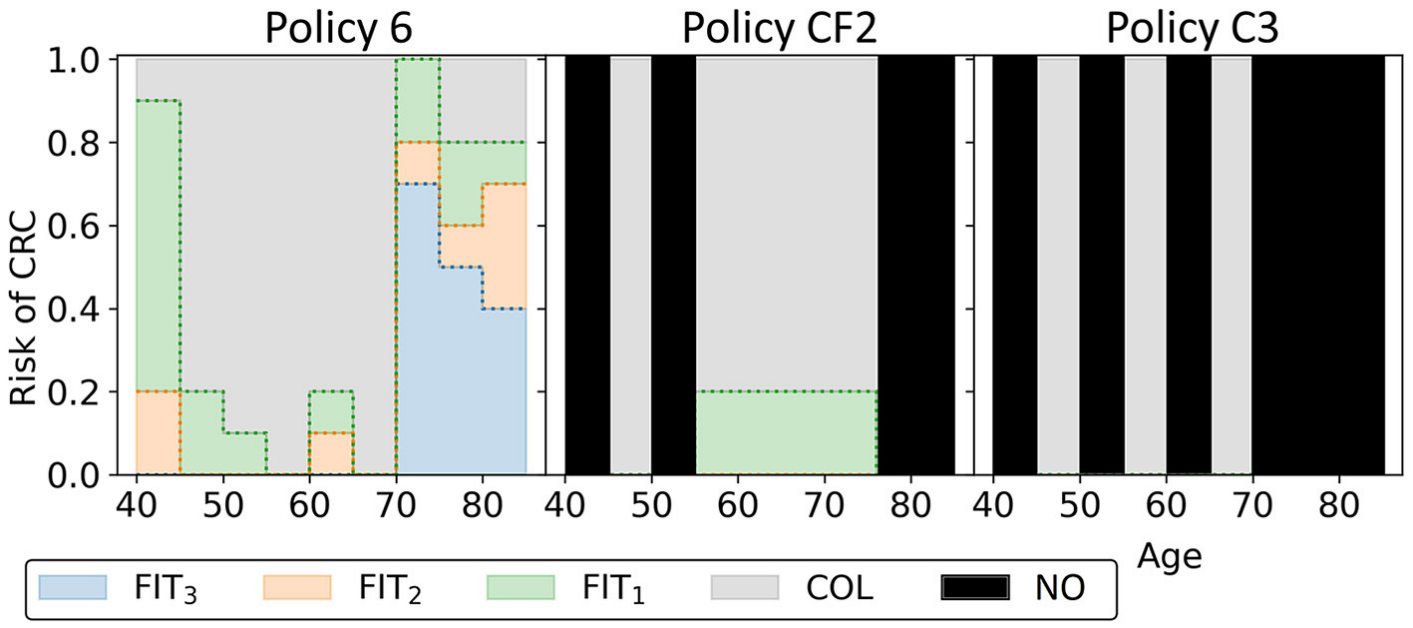
The three blue-red/green-red policies in Figure 10 are shown. Policy 6 is the cost-effective policy within the willingness-to-pay threshold in the approximation set for Experiment 2. Policies CF2 and C3 are the closest reference policies in terms of costs and QALYs (these policies are listed in Supplementary Table 3). For age groups with a black bar, reference policies do not offer screening.

To characterize the obtained approximation set, Figure 11 shows the cost-effective personalized policy in more detail (policy 6, marked blue-red in Figure 10), as well as two reference policies with comparable costs and QALYs (marked green-red in Figure 10). The reference policies initiate screening at age 45. Policy 6 prescribes screening before 45, but limits colonoscopy referrals by prescribing a high FIT-cutoff of 90 μg/g. The reference policies both stop screening at age 75. Policy 6 prescribes high cutoffs and long intervals from age 70. Since the algorithm is forced to design screening policies that start at age 40 and stop at age 85, we suspect that it tries to reduce the screening intensity by prescribing long intervals and high cutoffs for younger/older age ranges. Interestingly, the FIT-cutoffs at age ranges 55 and 65 in policy 6 are 0 μg/g, effectively resulting in a guaranteed referral for a colonoscopy regardless of the measured FIT-concentration. After such a colonoscopy, provided it was negative, screening is first halted for 5 years by design. We see that screening is then offered with higher cutoffs for another 5 years. Effectively, the colonoscopies are applied with a 10-year interval for most participants between these ages, in line with current USPSTF recommendations for colonoscopy-based screening and policy C3.

**Figure 12:**
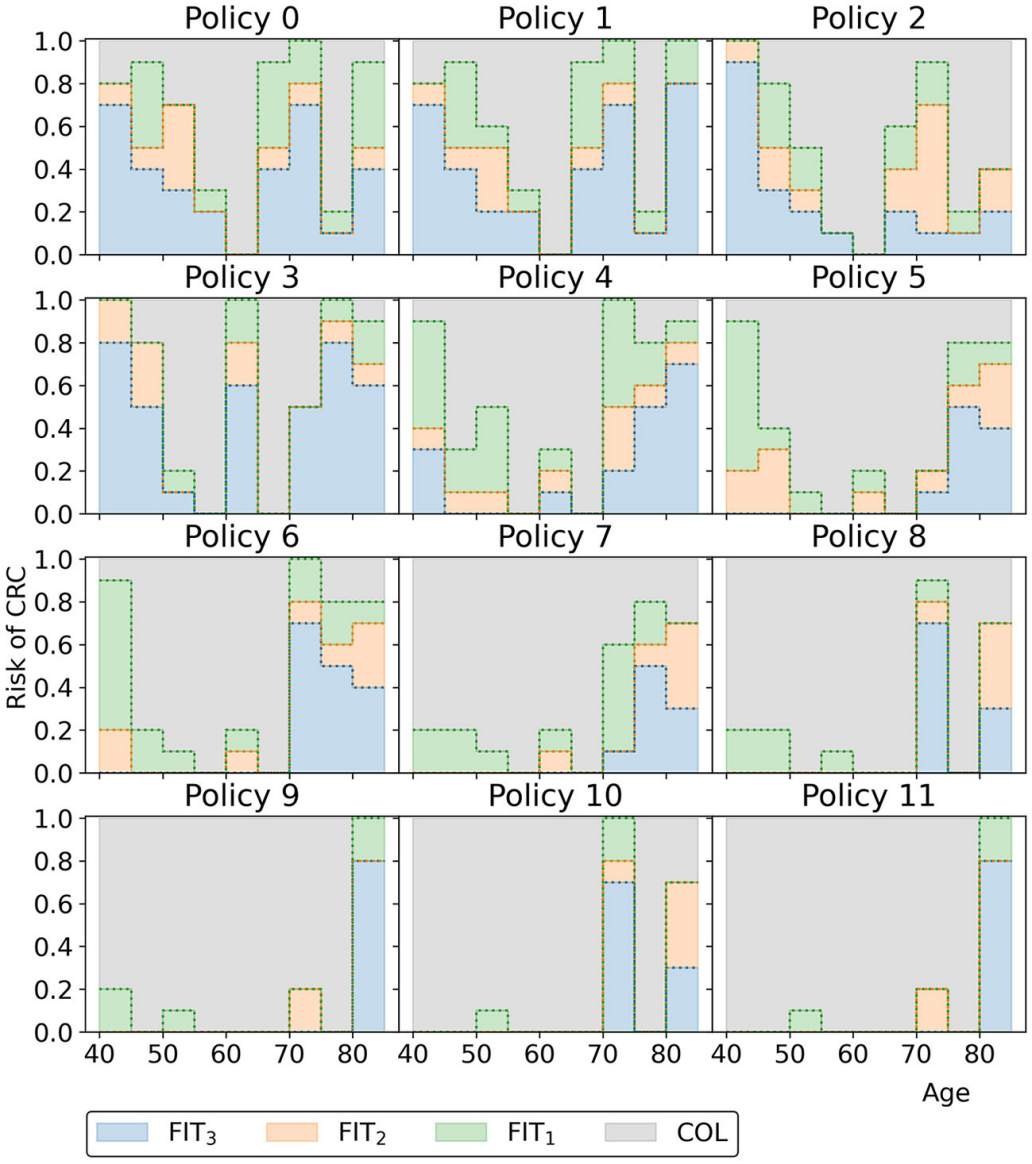
The policies that form the minimal representation of the approximation set of Experiment 2 as shown in Figure 10.

Figure 12 displays all policies that represent the blue approximation set in Figure 10 to observe the effect of decreasing or increasing the costs compared to policy 6. All policies offer intermittent colonoscopy and FIT-screening by prescribing at least one guaranteed colonoscopy and prescribing FIT-screening with higher cutoffs after a guaranteed colonoscopy with a negative result. The cheaper policies focus on FIT-screening during the ages 50 through 65. They apply higher cutoffs and longer screening intervals for other ages, limiting the screening intensity for those age ranges. This is a consequence of the lower risk of CRC for younger age ranges in general and the shorter life expectancy for older age ranges, effectively resulting in less life years to gain from screening. More expensive policies focus relatively more on colonoscopy screening (FIT-cutoffs of 0 μg/g) and decrease the cutoffs and the intervals first for those aged 40 and then for the 70+ age ranges. The most expensive policies prescribe multiple guaranteed colonoscopies, similar to the colonoscopy-based policies evaluated for the USPSTF.

### 3.3. Experiment 3: Optimizing Cutoffs

Experiment 3 has a smaller number of feasible policies compared to Experiment 2 because the action space was smaller. Nonetheless, the algorithm converged after 2,111 iterations, more than in Experiment 2. The third experiment was run in two phases. The first 505 iterations were run on a virtual machine managed by Erasmus Medical Center, the remaining 1,606 iterations on Bebop. The part run on Bebop was performed on 288 cores, enabling 286 concurrent model runs, with a total walltime of 65.5 h, and a computation time of 18,864 core hours. The evolutionary operators used 0.07% of the computation time, MISCAN-Colon used the remainder. The running times of Experiments 2 and 3 are incomparable because MISCAN-Colon was accelerated in between the two runs.

**Figure 13:**
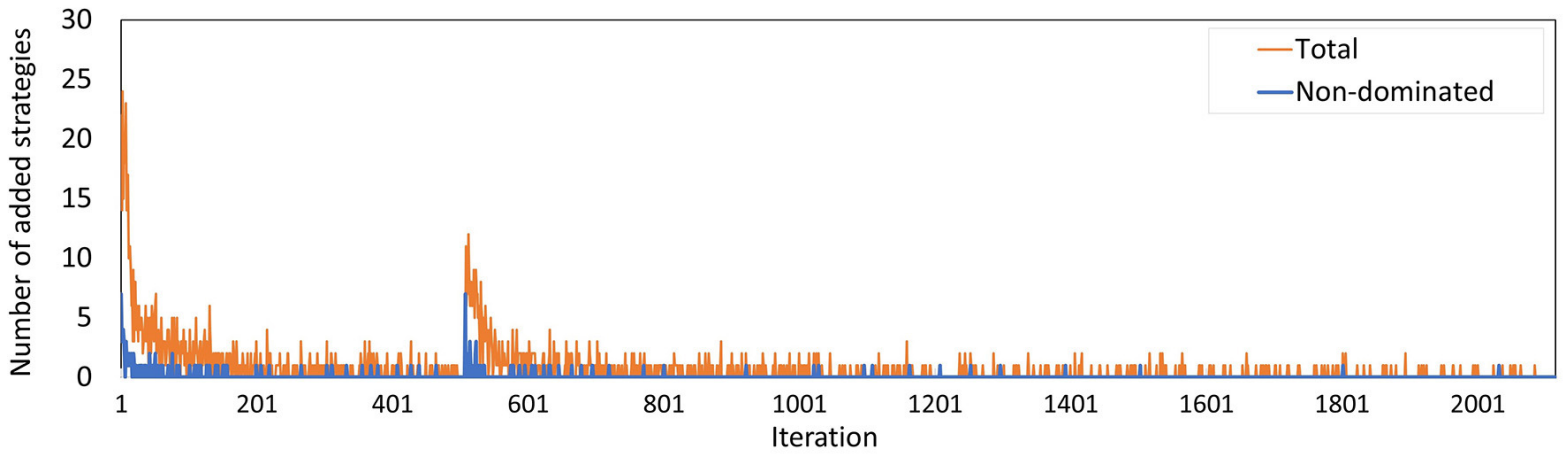
The number of policies added to the memory in each iteration for Experiment 3. The blue line counts all added policies, the orange line only those that are not dominated by (combinations of) other policies in the current memory. The latter are part of the minimal representation of the memory's approximation set. The peak at iteration 506 is caused by the different seeds used on the virtual machine and the Bebop.

The number of policies added to the memory per iteration (Figure 13) evolved along similar lines as in Experiment 2, where the minority of the policies added are not dominated by a combination of other policies, especially during the last few iterations. The peak at iteration 505 is caused by the changed random number stream for MISCAN-Colon when the runs were transferred from the virtual machine to the Bebop.

In Experiment 3, there were 13 policies to minimally represent the obtained approximation set (Figure 10). The figure shows that nearly all reference policies were dominated, except for two. The two exceptions are marked by white-green diamonds: triennial FIT for ages 45 through 70, and colonoscopy for age ranges 45 and 60 (policies F1 and C1 in Supplementary Table 3, resp.). Both policies quit screening relatively early whereas the personalized policies have a fixed stopping age of 85 by design. Disregarding these two reference policies, the QALYs of the obtained screening policies were up to 4.3% higher than the QALYs of the reference policies for similar costs.

**Figure 14:**
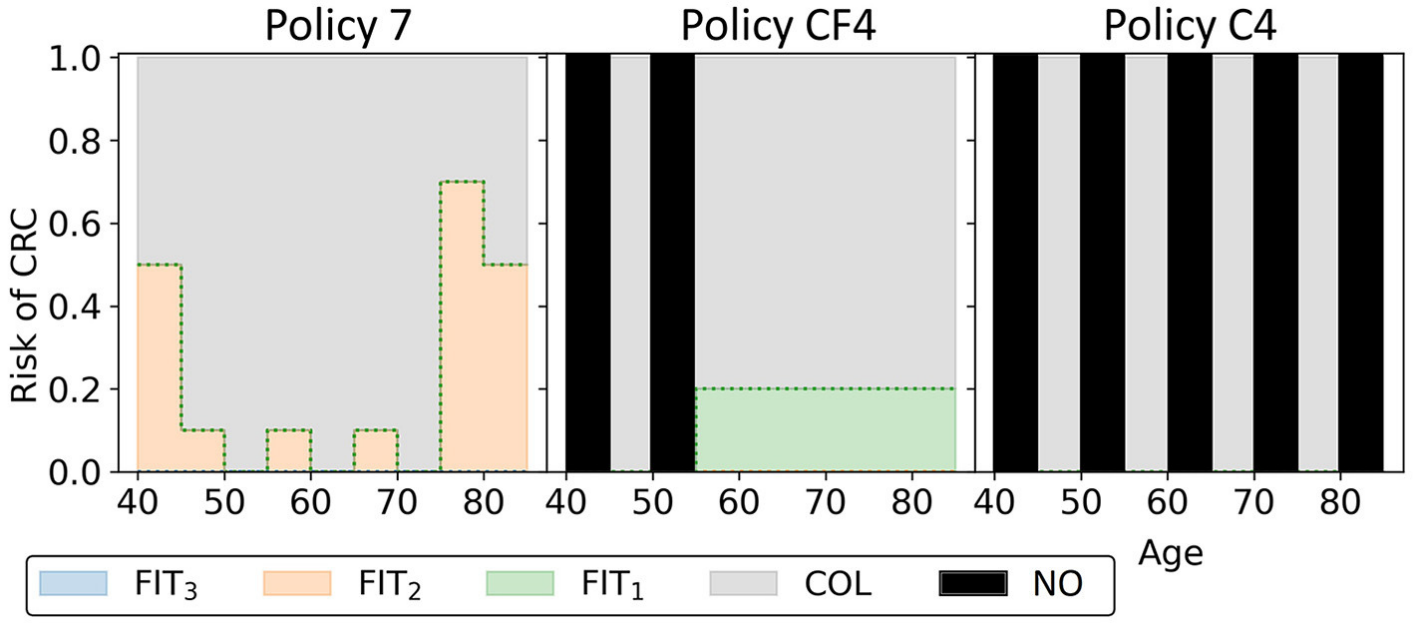
The three black-orange/green-orange policies in Figure 10 are shown. Policy 7 is the cost-effective policy within the willingness-to-pay threshold in the approximation set for Experiment 3. Policies CF4 and C4 are the closest reference policies in terms of costs and QALYs (see also Supplementary Table 3). For age groups with a black bar, no screening is offered.

In this experiment, the black-orange policy in Figure 10 was the cost-effective policy within the willingness-to-pay threshold (policy 7 in Figure 14). The two most similar reference policies with respect to costs and QALYs (marked green-yellow in Figure 10) commence screening at 45. Also, the screening intensity of policy 7 is low until age 45 as a cutoff of 50 μg/g is prescribed. The policies stop screening at age 80 or 85, though policy 7 has high cutoffs for colonoscopy referral from age 75. In between, policy 7 effectively prescribes 10-yearly colonoscopy for most participants, in line with US colonoscopy-based screening recommendations and policy C4.

**Figure 15:**
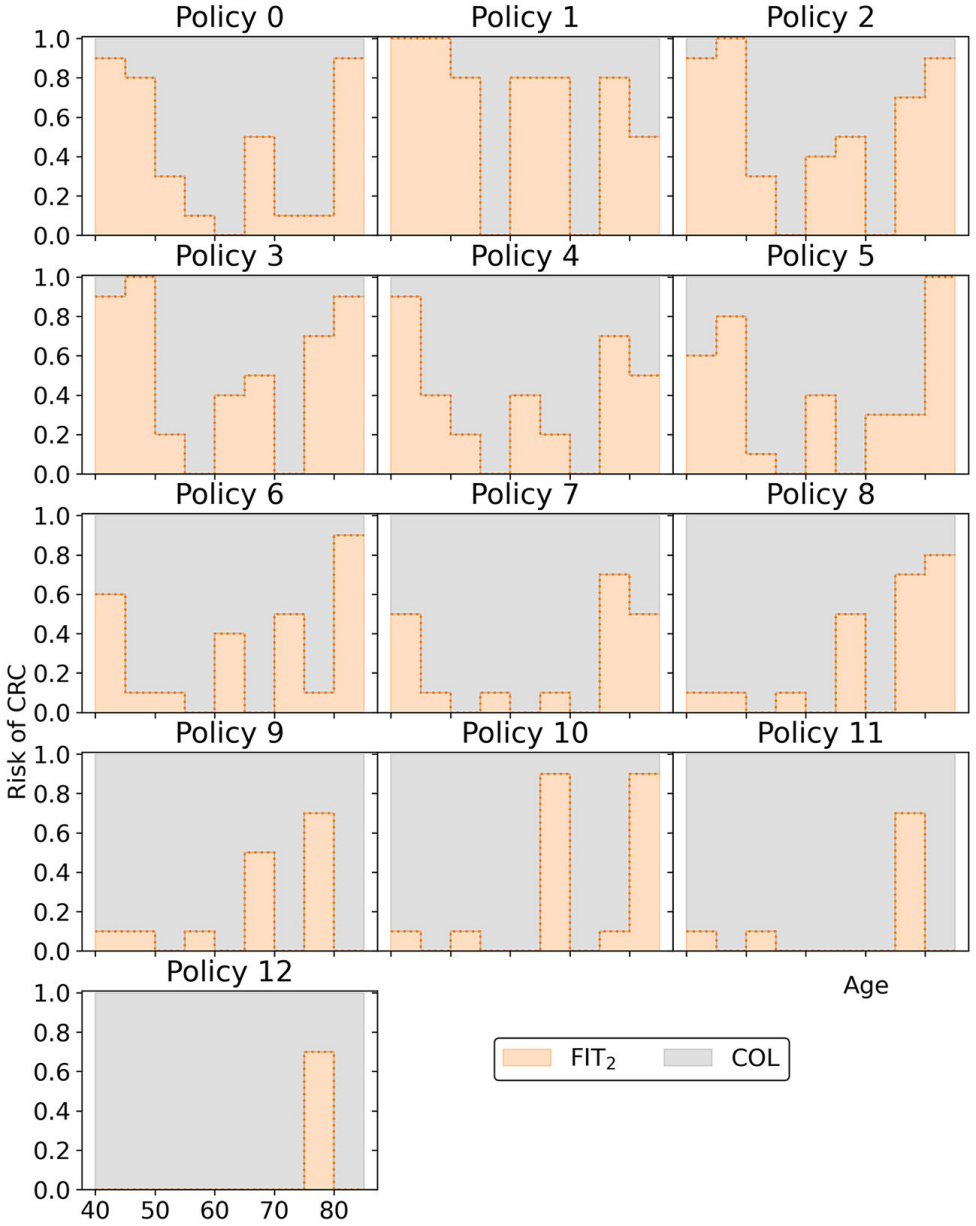
The policies that form the minimal representation of the approximation set of Experiment 3 as shown in Figure 10. Note that a cutoff at a perceived risk of 1.0 implies that participants with a FIT-concentration above 100 μg/g are referred for a colonoscopy.

Overall, the other policies in the minimal representation of the obtained approximation set (Figure 15) have patterns similar to the policies found in Experiment 2. Screening is primarily focused on the ages 50/55 through 75 for policies cheaper than policy 7. More expensive policies allow more screening in other age ranges, and the most expensive policies are more colonoscopy-based.

### 3.4. Comparing Experiments 2 and 3

Screening policies in Experiment 2 are more flexible as they have a larger variety in screening intervals compared to Experiment 3. However, with this flexibility, the number of feasible policies increases by a factor 10^13^ (see Supplementary Section 2). This means that the algorithm has a larger search space.

Figure 10 shows that the approximation set of Experiment 3 is dominated by that of Experiment 2. Figures 9, 13 show that the set was found in fewer iterations in the second experiment compared to the third. This suggests that it may be beneficial to increase the flexibility of the problem by increasing the action space, despite the increased search space.

## 4. Discussion

In this paper, we demonstrated the computational viability of designing and optimizing personalized FIT-based screening policies using an evolutionary algorithm. The algorithm combines with an advanced simulation model to evaluate the policies. The generated policies prescribed varying screening intervals or referral for a colonoscopy, based on a person's age and measured fecal haemoglobin concentrations. The evolutionary algorithm was used to generate a collection of personalized screening policies, also called an approximation set, that approximates the Pareto frontier, the set of policies with maximum benefits, measured in QALYs gained, for given costs. In our study, an established microsimulation model, MISCAN-Colon, was used to estimate the costs and QALYs of a screening policy.

We demonstrated the performance of the algorithm in three experiments. In the first, we used a relatively small problem instance with 1.5 million feasible policies. We calculated the exact optimal Pareto frontier and tested how well it was approximated by the algorithm. The algorithm could solve this instance to near-optimality, with an optimality gap of 0.007%.

The problem instances of the second and third experiments were too large to derive the exact Pareto frontier. We evaluated the performance of the evolutionary algorithm by (1) comparing the generated policies to a set of reference policies, previously evaluated with MISCAN-Colon in a decision analysis for the United States Preventive Services Task Force (USPSTF), in terms of costs and benefits and (2) assessing the face validity of the obtained policies. First, the generated personalized screening policies generally outperformed the reference policies in terms of costs and QALYs. For a given level of costs, the QALYs gained by the generated policies increased by 14% in Experiment 2 and 4.3% in Experiment 3. In Experiment 2, the computation time of the algorithm was four times shorter than the time of the enumeration process in Experiment 1, despite the 10^16^ times larger search space. This underscores the potential of personalized screening, and of the computational approach presented in this study.

Second, the obtained policies have several interesting features. The cost-effective policies allocated screening predominantly to the ages 50–70 or 45–70 through short intervals and low cutoffs for these ages. This is in line with currently implemented policies, which mostly prescribe screening to those aged 50–70 (Schreuders et al., 2015). Cheaper policies increased the intervals and cutoffs for the ages below 55 and above 65. This way, the algorithm narrows the focus of the policies to the ages 55–65 since policies are forced to apply screening from age 40 to 85 by design. More expensive policies expanded the age ranges with low cutoffs and short intervals. Remarkably, all policies guaranteed at least one colonoscopy to all participants by prescribing a FIT-cutoff of 0 μg/g for at least one age range. However, whenever a second guaranteed colonoscopy was offered, the interval from the previous colonoscopy was at least 10 years. This is in accordance with current US colonoscopy-based screening recommendations (Lin et al., 2021). The above observations support the algorithm's face validity, i.e., its ability to generate sensible policies.

In the second experiment, the policies prescribed a larger variety of screening intervals than in the third experiment, resulting in an increase of the search space by a factor 10^13^. Still, the approximation set found in Experiment 2 dominates the set found in Experiment 3. This suggests that a larger set of screening intervals is beneficial, despite the increased search space.

To the best of our knowledge, this is the first algorithm that optimizes personalized FIT-screening policies evaluated by an advanced microsimulation model. Whereas current methods impose strong Markov assumptions to evaluate generated policies, we evaluated them without such assumptions. The described algorithm is flexible: an individual's risk can be estimated by a variety of estimators, a wide range of actions can be incorporated in the action set, and custom age ranges to which policies apply may be considered. It may also be applied to other diseases when combined with a suitable simulation model that evaluates the costs and benefits of policies, as long as their screening program is based on a test with a quantitative test result. Examples include prostate specific antigen (PSA) based screening for prostate cancer or mammography screening for breast cancer. Such models are increasingly developed and our algorithm provides enough flexibility that it can be combined with many existing models.

The developed algorithm may be amenable for further improvement. First, it may be possible to enhance the evolutionary operators to search the space of screening policies more efficiently, for example by applying semi-random mutations directed by other simulation outcomes. Second, more fine-grained variations of the belief and action space may be considered, for example including information on prior colonoscopy results in addition to FIT-history, and the option to “stop screening”. Furthermore, additional user constraints may be applied to the policies generated by our algorithm, to facilitate easier implementation in practice. For example, it may not be desirable to prescribe guaranteed colonoscopies, or policy makers may want age-independent cutoffs for FIT-positivity for practical reasons. Decision scientists and policy makers should come up with a guideline of what features a policy requires for real-world implementation. We believe the computational framework presented in this paper is sufficiently flexible to incorporate such additional features.

As with any model, results from a microsimulation model are subject to uncertainty, and should be interpreted with caution. MISCAN-Colon was extensively validated in the past on randomized clinical trial data for screening, including fecal-based screening. However, the module for FIT-concentrations was a prototype model for which direct clinical validation was not possible in the scope of this study. It needs further development and validation when more data on the relation between FIT-concentrations and presence of lesions become available. On the other hand, the study shows that using a simpler but faster model could decrease the algorithm's computation time. In Experiments 2 and 3, 99.9% of the algorithm's running time was spent on simulation by MISCAN-Colon, despite parallel computations. However, this may be at the cost of decreased accuracy in the evaluation of the policies.

To conclude, we demonstrated a potential method for identifying optimized personalized screening policies while evaluating them with established simulation models from practice. This moves the field a step closer to implementing personalized screening in practice.

## Data Availability Statement

An implementation of the algorithm in Python is available at https://gitlab.com/luukvandEMC/ea_personalized_screening. It includes a fictive dataset similar to the benchmark set of Experiment 1.

## Author Contributions

LD developed the algorithm, performed the analysis, and wrote the manuscript. RM, RS, and IL-V contributed as supervisors. RM also helped develop the module to simulate fecal occult blood loss. NC and JO conducted the experiments on the High Performance Computer. All authors contributed to the manuscript and approved the submitted version.

## Funding

The microsimulation analysis was supported by Grant U01-CA199335 and Grant U01-CA253913 from the National Cancer Institute (NCI) as part of the Cancer Intervention and Surveillance Modeling Network (CISNET). The content is solely the responsibility of the authors and does not necessarily represent the official views of the National Institutes of Health. The work was supported in part by the U.S. Department of Energy, Office of Science, under contract (No. DE- AC02-06CH11357).

## Conflict of Interest

The authors declare that the research was conducted in the absence of any commercial or financial relationships that could be construed as a potential conflict of interest.

## Publisher's Note

All claims expressed in this article are solely those of the authors and do not necessarily represent those of their affiliated organizations, or those of the publisher, the editors and the reviewers. Any product that may be evaluated in this article, or claim that may be made by its manufacturer, is not guaranteed or endorsed by the publisher.
