# Supplementary material for: An Evolutionary Algorithm to Personalize Stool-Based Colorectal Cancer Screening
Source: Front Physiol. 2022 Jan 26;12:718276. doi: 10.3389/fphys.2021.718276 (PMC8826712; doi:10.3389/fphys.2021.718276)
Supplement: Supplementary file 1 [file Data_Sheet_1.pdf]

Supplementary Material

1 MODEL ASSUMPTIONS OF MISCAN-COLON

MISCAN-Colon is a microsimulation model: it simulates individual life histories from birth to death. Some individuals develop colorectal cancer (CRC). In the model, all cancers start as an adenoma and develop through the conventional adenoma-carcinoma pathway, shown in Figure S1. In this pathway, an adenoma is small ($\leq$ 5 mm) initially. Then it may grow to a medium-sized (6-9 mm) or large adenoma ($\geq$ 10 mm). Such adenomas are screen-detectable but do not cause any symptoms. Adenomas can be progressive or non-progressive. Only progressive adenomas can develop into CRC.

When CRC develops, at first it is preclinical. This means that the cancer does not cause clinical symptoms. It may progress from stage I through stages II, III and IV or be diagnosed through symptoms in any of those stages. Preclinical cancers are screen-detectable. Once a cancer becomes clinical, either diagnosed by symptoms or by screen-detection, it is treated. Survival depends on stage and anatomic location of the diagnosed CRC. During the simulation, individuals may also die of competing causes of death.

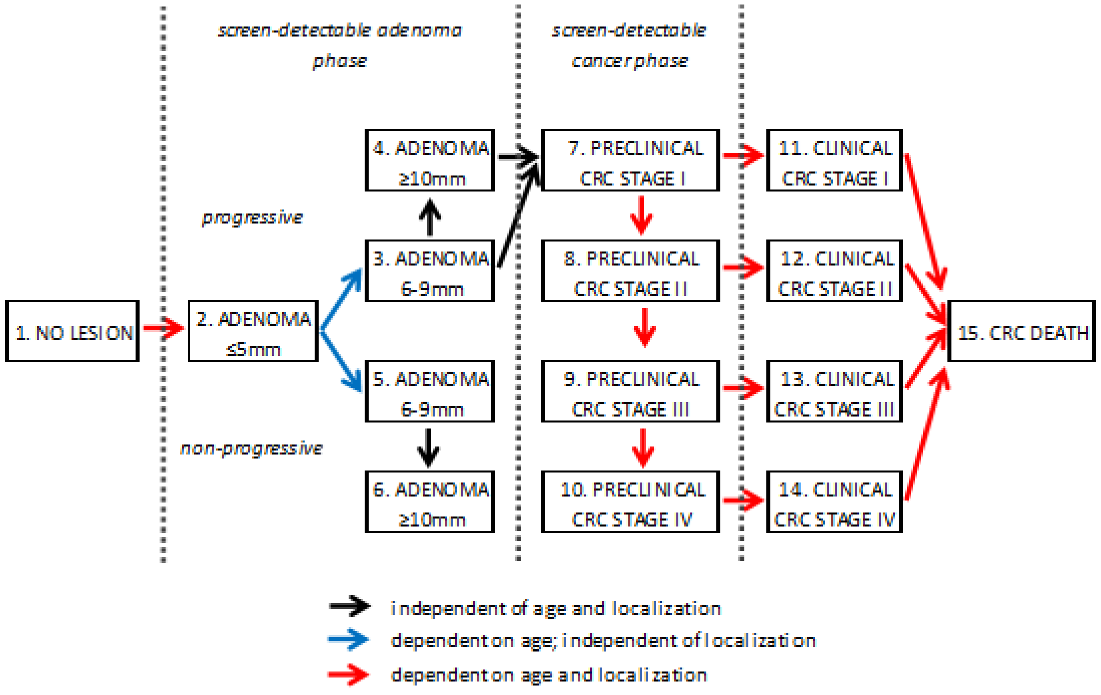

Figure S1. The pathway from adenoma to cancer death as simulated in MISCAN-Colon.

In the screening component of MISCAN-Colon, screen-participants undergo a screening test, FIT in this study. Whether all simulated individuals participate depends on the assumption on screening participation. If the screening test is positive, participants are referred for a follow-up test, which is a colonoscopy in this study. In case of a positive follow-up, participants leave the screening program and start a surveillance program. This prescribes a regular colonoscopy where the interval depends on the findings. Surveillance is stopped at a fixed age. Screening has benefits, such as early detection and removal of adenomas or earlier

Table S1. Model characteristics and screening assumptions as implemented in MISCAN-Colon for this analysis.

**General model characteristics**	
Model type	Microsimulation model
Simulation time step	Continuous time
**Screening policy**	
Screening test	FIT
Screening interval	1, 2 or 3 years
Follow-up test (after postive screening test)	Colonoscopy
Screening participation	100%
**FIT characteristics**	
Sensitivity and specificity depend on the chosen cutoff and the mathematical model presented below.	
**Colonoscopy characteristics (per person)**	
Specificity	100%
Sensitivity for small adenomas	75%
Sensitivity for medium-sized adenomas	85%
Sensitivity for large adenomas / CRC	95%
Reach	94%
Fatal probability for adenoma removal	0.001%
**Surveillance program (after positive follow-up test)**	
Surveillance test	Colonoscopy
Surveillance interval by finding:	
At least one large adenoma found	3 years
Otherwise	5 years
Surveillance stopping age:	
Experiment 1	76
Experiments 2, 3 and reference strategies	86

diagnosis of preclinical cancers, resulting in life years gained. It also comes with harms as screening tests are unable to distinguish between progressive and non-progressive adenomas or individuals die of other causes before they would have died of a detected CRC.

Table S1 shows the model characteristics and screening assumptions of MISCAN-Colon that apply to all three experiments.

In our study, FIT-concentrations are simulated quantitatively and the test sensitivity and specificity depend on the chosen cutoff. Our preliminary module to simulate FIT-concentrations has a linear mixed-effects model (GLMM) structure. It assumes a zero-inflated negative binomial distribution, conditional on the characteristics $\boldsymbol{x}_n$ of individual n (age, gender and adenoma/CRC stage). These characteristics may change over time. The structure of the module is as follows. The FIT-concentration C equals 0 with a probability φ which depends on the individual's (highest) adenoma/CRC stage x_STAGE. We use three different values for φ: one for (1) healthy individuals and those with non-advanced adenomas (small or medium-sized), (2) those with advanced adenomas (large) and (3) those with a preclinical cancer.

$$C|\boldsymbol{x}_n \sim \begin{cases} 0, & \text{with probability } \varphi(x\_STAGE) \\ NB(C|\boldsymbol{x}_n), & \text{otherwise.} \end{cases}$$

Table S2. Overview of the variables in the model for Hb concentrations and their values as used in this study.

Symbol	Description	Value
$\varphi(H)$	Probability of inflated zero for healthy + non-advanced adenoma stages	0.840
$\varphi(AA)$	Probability of inflated zero for advanced adenoma stage	0.644
$\varphi(PC)$	Probability of inflated zero for preclinical stages	0.032
$b_{\text{INTERCEPT}}$	Intercept	1.481
b_{AGE}	Weight of age	0.0181
b_{MALE}	Weight of gender	0.2832
b_{AA}	Weight of advanced adenoma stage	0.609
b_{PC}	Weight of preclinical stages	8.74
θ	Dispersion factor	0.262
σ	Standard deviation of individual risk factor distribution	0.1120

The negative binomial probability distribution is the following:

$$NB(C|\boldsymbol{x}_n) = \frac{\Gamma(C+\theta)}{C!\Gamma(\theta)} * \left(\frac{\theta}{\theta+\mu_n}\right)^{\theta} * \left(\frac{\mu_n}{\theta+\mu_n}\right)^{C},$$

with θ the dispersion factor, constant throughout the algorithm, and $\Gamma(.)$ the gamma function. The mean μ_n is defined by

$$\mu_n = \mu(\boldsymbol{x}_n) = \exp\{b_{\text{INTERCEPT}} + \gamma_n + b_{\text{AGE}} * x\_\text{AGE} + b_{\text{MALE}} * I(x\_\text{GENDER} = \text{MALE}) + b_{\text{AA}} * I(x\_\text{STAGE} = \text{AA}) + b_{\text{PC}} * I(x\_\text{STAGE} = \text{PC})\}.$$

b_i represents the weight of each of the properties $x_i \in \boldsymbol{x}_n$, $I(.)$ is the indicator function. ”AA” stands for advanced adenomas, ”PC” for prelicinal cancer stages. γ_n is an individual risk factor, a random value that is drawn for all participants individually and remains constant during their lives. It is responsible for the correlation between the Hb concentrations obtained in the same individuals. Its distribution is $\gamma_n \sim \mathcal{N}(0, \sigma)$.

Table S2 gives an overview of the calibrated parameters.

2 NUMBER OF FEASIBLE POLICIES IN THE EXPERIMENTS

The three experiments in this study use three different problem instances. We show how the number of feasible policies can be calculated for each instance.

First we consider the benchmark instance in Experiment 1. Consider a single age range τ. If our action set included one screening interval only, i.e. $\mathcal{A} = \{FIT_1, COL\}$, there were 5 unique screening policies for this age range: since $\mathcal{R} = \{0, 0.125, 0.25, 0.375, 0.5\}$, the single policy bound β_1 can take five values. When we add an extra interval FIT_2 to the action set, we need to account for the test order assumption. If $\beta_2 = 0$, β_1 can take any of the five values in $\mathcal{R}$. If $\beta_2 = 0.125$, β_1 can take the four values equal or greater than 0.125. If $\beta_2 = 0.25$, β_1 can take the three values equal or greater than 0.25. This leads to $5 + 4 + 3 + 2 + 1 = 15$ options. When adding a third screening interval FIT_3 to the action set, the above calculation applies when $\beta_3 = 0$. If $\beta_3 = 0.125$, there are $4 + 3 + 2 + 1 = 10$ policies.

The above leads to $15 + 10 + 6 + 3 + 1 = 35$ options for the considered age range τ. Experiment 1 has four screen-eligible age ranges, each with an independent screening policy, resulting in $35^4 = 1,500,625$ unique policies.

Table S3. An overview of the used reference policies evaluated for the United States Preventive Services Task Force by Knudsen et al. (2020). There are four types. Policies marked by Cx prescribe a colonoscopy at specific ages. Policies marked by Fx prescribe a regular FIT between the start and stop age. CFx policies prescribe a single colonoscopy followed by annual FIT. FCx policies prescribe five years of annual FIT, followed by a 10-yearly colonoscopy. All prescribed FITs have a cutoff of 20 μg/g.

	**Colonoscopy Ages**	**FIT Start age**	**Stop age**	**Interval**
C1	45, 60			
C2	45, 60, 75			
C3	45, 55, 65			
C4	45, 55, 65, 75			
C5	45, 55, 65, 75			
C6	45, 50, 60, 65, 70			
C7	45, 50, 60, 65, 70, 75			
C8	45, 50, 60, 65, 70, 75, 80			
C9	45, 50, 60, 65, 70, 75, 80, 85			
F1		45	70	3
F2		45	80	3
F3		45	75	2
F4		45	80	2
F5		45	85	2
F6		45	75	1
F7		45	80	1
F8		45	85	1
CF1	45	55	70	1
CF2	45	55	75	1
CF3	45	55	80	1
CF4	45	55	85	1
FC1	50, 60, 70	45	49	1
FC2	50, 60, 70, 80	45	49	1

Generalizing the above, when an action set includes three screening intervals, we calculate the number of feasible policies in the problem instance by

$$\left(\sum_{k=1}^{|\mathcal{R}|} \sum_{j=1}^{k} j \right)^{|\mathcal{T}|} .$$

Experiment 2 also uses an action set with three screening intervals. With $|\mathcal{R}| = 11$ and $|\mathcal{T}| = 9$, it has $286^9 = 1.28e22$ feasible policies.

Experiment 3 uses a simple action set with one screening interval. It includes $|\mathcal{R}|^{|\mathcal{T}|} = 11^9 = 2.3e9$ feasible policies.

3 REFERENCE POLICIES

Table S3 gives an overview of the reference policies used in Experiments 2 and 3.

REFERENCES

Knudsen, A. B., Rutter, C. M., Peterse, E. F. P., Lietz, A. P., Seguin, C. L., Meester, R. G. S., et al. (2020). Colorectal cancer screening: A decision analysis for the u.s. preventive services task force
